# A Comprehensive Sampling Study on SARS-CoV-2 Contamination of Air and Surfaces in a Large Meat Processing Plant Experiencing COVID-19 Clusters in June 2020

**DOI:** 10.1097/JOM.0000000000002785

**Published:** 2023-01-11

**Authors:** Myrna M.T. de Rooij, Reina S. Sikkema, Martijn Bouwknegt, Yvette de Geus, Kamelia R. Stanoeva, Sigrid Nieuwenweg, Adriana S.G. van Dam, Ceder Raben, Wietske Dohmen, Dick Heederik, Chantal Reusken, Adam Meijer, Marion P.G. Koopmans, Eelco Franz, Lidwien A.M. Smit

**Affiliations:** From the Institute for Risk Assessment Sciences, Utrecht University, Utrecht, the Netherlands (M.M.T.d.R., W.D., D.H., L.A.M.S., Y.d.G., S.N., and C.R.); Department of Viroscience, Erasmus MC, Rotterdam, the Netherlands (R.S.S., M.P.G.K.); Vion Food Group, Boxtel, the Netherlands (M.B.); Center for Infectious Disease Control, National Institute for Public Health and the Environment (RIVM), Bilthoven, the Netherlands (K.R.S., C.R., A.M., and E.F.); European Public Health Microbiology Training Programme, European Centre for Disease Prevention and Control, Stockholm, Sweden (K.R.S.); Municipal Health Services GGD Hart voor Brabant, 's-Hertogenbosch, the Netherlands (A.S.G.v.D.).

**Keywords:** air, environmental transmission, meat processing plant, occupational health, SARS-CoV-2, surfaces

## Abstract

We aimed to assess SARS-CoV-2 contamination of air and surfaces to gain insight into potential occupational exposure in a large meat processing plant experiencing COVID-19 clusters. **Methods:** Oro-nasopharyngeal SARS-CoV-2 screening was performed in 76 workers. Environmental samples (*n* = 275) including air, ventilation systems, sewage, and swabs of high-touch surfaces and workers' hands were tested for SARS-CoV-2 RNA by real-time quantitative polymerase chain reaction. **Results:** Twenty-seven (35.5%) of the (predominantly asymptomatic) workers tested positive with modest to low viral loads (cycle threshold ≥ 29.7). Six of 203 surface swabs, 1 of 12 personal air samples, and one of four sewage samples tested positive; other samples tested negative. **Conclusions:** Although one third of workers tested positive, environmental contamination was limited. Widespread SARS-CoV-2 transmission via air and surfaces was considered unlikely within this plant at the time of investigation while strict COVID-19 control measures were already implemented.

## Abstract 2

Comprehensive environmental sampling showed limited SARS-CoV-2 contamination of air and surfaces in a meat processing plant, despite a large proportion of workers testing SARS-CoV-2 positive. A strict COVID-19 policy was in place, suggesting that SARS-CoV-2 transmission may be controlled in meat processing plants by enforcing appropriate preventive and mitigation measures.

**LEARNING OUTCOMES**

After completion of this educational activity, the learner will:
• have a deeper understanding of the different environmental sampling approaches to comprehensively assess contamination by infectious agents of air and surfaces
• be more aware of the working conditions of meat processing plant workers and the associated increased transmission risks making these occupational populations vulnerable for infectious diseases
• better appreciate the need for a multidisciplinary approach to properly assess transmission routes of infectious agents by combining insights from epidemiological studies as well as experimental and modeling research with empirical exposure assessment

Since the beginning of the COVID-19 pandemic in early 2020, meat processing plants have been identified as SARS-CoV-2 infection hotspots across Europe, Australia, and the Americas.^1,2^ Essential services/industries like the food industry were exempted from lockdown and remained active. Obviously, this required implementation of COVID-19 mitigation measures in meat processing facilities that were continuously updated based on progressive insight. Still, many SARS-CoV-2 outbreaks occurred including uncontrollable ones that forced shutdown of the affected facilities.^1,2^ A combination of several factors may have caused meat processing plants to be SARS-CoV-2 infection hotspots, including operational practices (eg, high density of workers, enhanced breathing and yelling due to the physically intense work, and noisy environment), societal and/or economic factors (eg, migrant workers sharing housing and transportation), and the climate conditions inside the production rooms.^1–3^

The probable relevance of climate conditions was emphasized by both experimental research and epidemiological studies showing COVID-19 clusters mainly occurring among workers operating in cooled production areas. The low temperature, which is in place to ensure food safety, combined with presence of air recirculation systems to reduce energy use, is considered advantageous for persistence and circulation of SARS-CoV-2 in air.^4–8^ Presumed importance of airborne transmission was substantiated by several epidemiological studies^9–12^ on the course of outbreaks in multiple meat processing plants, showing associations with ventilation and airflow. Besides low temperatures being advantageous for airborne transmission, it can also facilitate fomite transmission (touching a contaminated surface and then transferring virus to facial mucosa) as experiments showed prolonged viability of SARS-CoV-2 on surfaces with cooler temperatures.^5,8,13^ Transmission control under environmental conditions that favor SARS-CoV-2 persistence is obviously more difficult and requires careful evaluation of the potential role of environmental transmission, for example, via air and surfaces. In this context, a multidisciplinary approach is needed, combining insights from epidemiological studies, experimental and modeling research,^14^ and empirical exposure assessment to properly assess transmission routes.^15^ Environmental sampling studies have been performed in diverse indoor environments,^16^ mainly hospitals, but are lacking still for meat processing plants.

An increased incidence of SARS-CoV-2 infections was notified among workers in cooled production rooms of a Dutch high-throughput pig meat processing plant by the end of May 2020. This elevated SARS-CoV-2 incidence among workers was in contrast to the low regional and national incidence at that time in the Netherlands. In the slaughterhouse, the COVID-19 policy already in place was sharpened with stricter measures and supervision on compliance was intensified. Starting early June 2020, we conducted a study to assess potential SARS-CoV-2 transmission via air and surfaces in this plant, in the context of COVID-19 measures in place. Comprehensive environmental sampling was performed simultaneously with voluntary screening for SARS-CoV-2 RNA among employees.

## METHODS

**FIGURE 1:**
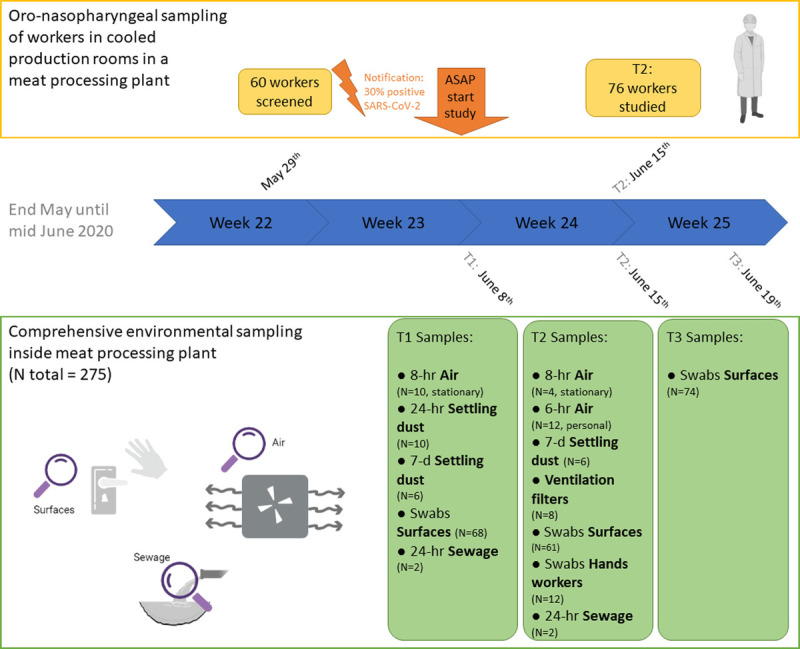
Overview at a glance of the study design.

Details and pictures of study setting, sampling methods, and laboratory procedures are provided in the Supplemental Digital Content, http://links.lww.com/JOM/B257. See Figure 1 for an overview at a glance of the study design.

### Investigated Slaughterhouse

Investigations were performed at a high-throughput pig slaughterhouse in the Netherlands. The production process can be divided into two parts: (1) process from live animals until halved carcasses and (2) process where carcasses are further sectioned, processed, and packed. The latter is performed in two large cooled production rooms (temperature, 5°C to 9°C): a cutting room of 9000 m^3^ and a deboning room with a packaging area of 10,800 m^3^. The number of persons working in the abattoir during each shift is around 850, of whom 600 are working in cooled production rooms (215 in the cutting room, 385 in the deboning room/packaging area). The abattoir is in production 6 days a week (Monday to Saturday), and per day, two consecutive shifts are scheduled (morning shift and afternoon/evening shift, with the exception of Saturday with solely a morning shift). In general, workers are scheduled to work 1 week in the morning shift and the next week in the afternoon shift in pools with stable composition. Workers typically have a fixed job task and operate at the same position along the processing line. There is a strict separation between the first (non-cooled) and second (cooled) part of the production process regarding personnel, areas accessible to personnel, materials, and clothing.

Cooled production rooms are ventilated by a system comprising two-stage filtering. The first stage includes a filter for larger particles (ISO 16890 Coarse 50%); the second stage includes a filter for smaller particles (ISO 16890 ePM_10_ 80% and ISO 16890 ePM_2.5_ 70%). Air is largely being recirculated, with minimally passive air refreshment through, for example, open inner doorways and corridors. Each day after production, a rigorous multistage cleaning procedure is followed involving wetting from top to bottom with a mix of cleaning/disinfecting agents including chlorine-based agents. Since June 2020, fogging was also performed each Sunday with hydrogen peroxide and lactic acid as active substances.

Screening for SARS-CoV-2 RT-qPCR (real-time quantitative polymerase chain reaction) status among a random selection of voluntarily participating abattoir workers on May 29 showed an especially high prevalence among workers operating in cooled production rooms: 41% in the cutting room (9/22), 32% in the deboning room (6/19), and 16% in the packaging area (3/19) versus 0% (0/45) in other sections. From March 2020, initial COVID-19 measures were implemented involving prevention of close contact between workers (separation of work shifts and breaks in time, workplace modifications) and increased focus on hand hygiene at entry of the premises and in non-production locations. From the start of June, additional measures were implemented involving intensified cleaning and disinfection procedures (including air treatment by fogging every Sunday with hydrogen peroxide and lactic acids), a triage based on symptoms (questionnaire and interview) of all individuals entering, and contact reductions while commuting.

### Sampling Strategy

Environmental sampling was started as soon as possible after notification of an increased SARS-CoV-2 incidence among the workers; environmental samples were collected at three time points in June 2020 (T1, June 8; T2, June 15; T3, June 19). SARS-CoV-2 RT-qPCR screening of a random selection of workers by oro-nasopharyngeal sampling was performed at T2 when confirmation of the Medical Research Ethics Committee was received. Screening based on sewage sampling was performed at T1 and T2 (not at T3 because of logistics).

To assess airborne SARS-CoV-2, we performed sampling of air, settling dust and filters of the ventilation system. To assess potential contamination of surfaces, swabs were collected from surfaces that were expected to be touched frequently as well as the hands/gloves of workers. At T1, the purpose of environmental sampling was to gain broad insight into potential environmental SARS-CoV-2 RNA presence in the various areas either in air or on surfaces. Stationary air sampling was performed at potential hotspots based on workers' density and ventilation characteristics in both production rooms. Environmental swabs were used to sample a selection of various high-touch surfaces present throughout the facility. At T2, focus was on personal air sampling during the shift of workers participating in SARS-CoV-2 oro-nasopharyngeal screening combined with swabbing of their hands/gloves. Environmental swabs were taken from high-touch surfaces not yet sampled. At T3, environmental swabs were collected from same and similar high-touch surfaces identified to be relevant at T2. Throughout the study, strict safety and hygienic procedures were followed to prevent infection and contamination. Field blanks of all sample types were collected as a control.

### Screening and Scoring

Sewage samples (two tubes of 50-mL 24-hour flow-dependent composite sample) were collected as described previously^17^ at both T1 and T2 in collaboration with the external water treatment plant located at the facility. At T2, in collaboration with the municipal health services (GGD), oro-nasopharyngeal swabs were collected from persons working at the cooled production rooms before and after the shift (minimum working time, 6.5 hours). The GGD team consisted of multiple experienced testers for time efficiency; workers were randomly assigned to a tester per test moment. Questionnaires were collected including items on health status, contacts, and working and living conditions. Workers participated on a voluntary basis, and written informed consents were obtained. Each worker received 40 euros for participation.

Workers were scored on SARS-CoV-2 transmission relevant behavior and personal protective measures by means of scoring cards by fieldworkers. To gain an overall impression of wearing surgical masks (categorized: covering nose and mouth, covering mouth, or not wearing), a minimum of 45 persons in both production rooms were scored. In addition, 5-minute observations of workers performing their job tasks were performed to register wearing of personal protective measures and physical distancing (both for longer durations, eg, conversations and solely passing).

### Sampling Air and Surfaces

Air sampling methodology was similar as described previously by de Rooij et al.^18^ In short, a filter-based technique was used to sample inhalable dust—airborne particles small enough to enter the respiratory tract. For stationary air sampling, sampling heads were attached onto a pole at a 1.50-m height (average breathing height of humans). Personal air sampling was performed by attaching the sampling head within the breathing zone of the worker. Stationary 6-hour sampling was performed in both production rooms. At T1, sampling was performed at five sites per room. At T2, stationary sampling was performed at two sites per room; the remainder of sampling equipment was used for personal sampling. Of the workers participating in oro-nasopharyngeal screening, 12 workers (six per room) were selected to participate in personal air sampling. Personal air sampling was performed from the beginning until the end of the worker's shift, resulting in 6- to 8-hour measurements. Sampling of settling dust in production rooms and the canteen was performed by using electrostatic dustfall collectors, which contain electrostatic cloths placed in a disposable holder, as described previously.^19^

Sampling of the ventilation system was performed at T2 for both production rooms. Per room, one filter of each type (Coarse 50% and ePM10 80%/ePM2.5 70%) was collected from their respective grid. These filters had been placed in August 2019.

Swabs of high-touch surfaces were collected in the production rooms and in all other areas workers have access to (eg, canteen area, locker room, toilets). Per time point, at least 60 surface swabs were taken throughout these areas. Swabs of hands, or gloves if worn, of the 12 workers participating in the personal air sampling were collected during their midshift break.

### Sample Processing and Laboratory Procedures

Samples were stored after collection at 4°C. At the end of the working day, samples were transported to the laboratory to be processed within 24 hours after collection at biosafety level 2 conditions. From oro-nasopharyngeal samples, total nucleic acid was extracted using a MagNA Pure 96 with total nucleic acid small volume kit (Roche). Thereafter, samples were tested for the presence of SARS-CoV-2 RNA using RT-qPCR, targeting the E gene and the RdRP gene with detection limits at 3.2 and 3.7 RNA copies/reaction, respectively.^20,21^ A worker was defined positive if at least one of the two genome targets tested positive in one or both swabs.

The other samples (non-standard sample types) were processed in a research laboratory; RNA extraction was performed using an in-house method using AMPure beads.^22^ These samples were tested for the presence of SARS-CoV-2 RNA using RT-qPCR, targeting the E gene (detection limit, 3.3 RNA copies/reaction).^20,21^

## RESULTS

### Screening

**FIGURE 2:**
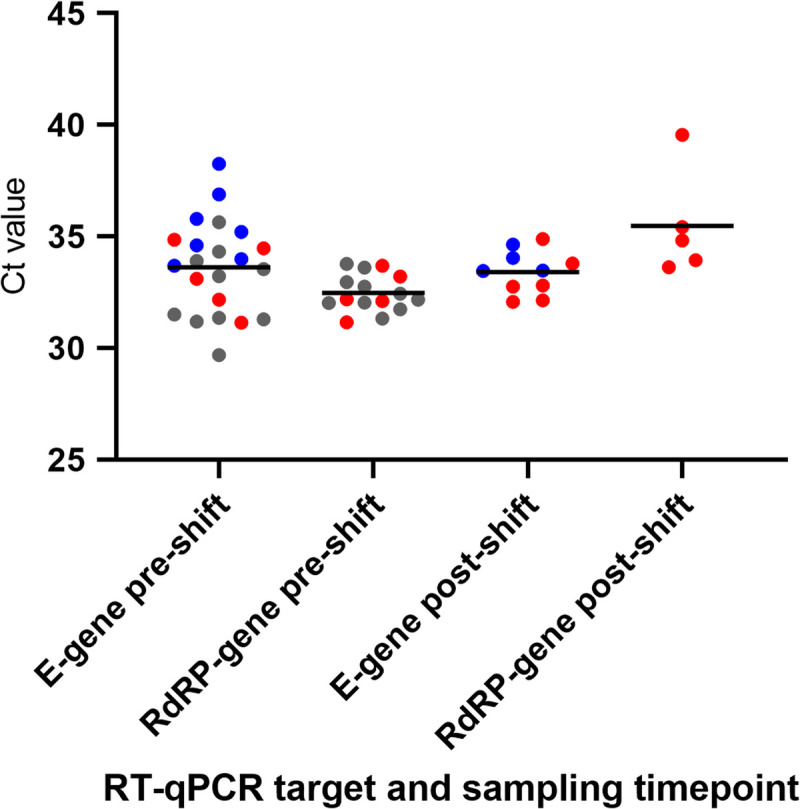
Column scatter plot showing distribution of Ct values by gene target and moment of sampling (pre-shift, post-shift) detected in oro-nasopharyngeal swabs from 27 meat processing workers who tested SARS-CoV-2 RNA positive on June 15, 2020. Each dot represents a positive oro-nasopharyngeal test result. The color represents the category to which the worker belongs: red dots indicate six employees who were positive at both sampling moments (pre-shift and post-shift) for one or two target genes; blue dots indicate 11 employees who were positive for one target gene and one sampling moment; gray dots indicate 10 employees who were positive for both target genes pre-shift only. The horizontal bar indicates the mean Ct value, which was computed by taking the arithmetic mean of the Ct values per gene target and moment of sampling.

Of the 81 workers invited, 76 (94%) participated in the oro-nasopharyngeal SARS-CoV-2 screening performed at T2. One worker solely participated in the pre-shift sampling round (sample tested negative). In total, 27 workers (35.5%) tested positive for SARS-CoV-2 RNA (Table 1). Of the cutting room workers, 21% tested positive versus 50% of the deboning area workers. Most workers were Polish or Romanian; in both groups, 40% tested positive. For six persons (22% of the test-positive cases), SARS-CoV-2 RNA was detected in both pre- and post-shift swabs. Seventeen workers tested positive pre-shift and negative post-shift, whereas only four workers tested negative pre-shift and positive post-shift. Cycle threshold (Ct) values ranged between 29.7 and 38.3 for the E gene and between 31.2 and 39.6 for the RdRp gene (Fig. 2), corresponding to modest to low viral loads. Of the 76 workers, 74 (97%) filled in the questionnaire. The two workers who did not return the questionnaire tested SARS-CoV-2 negative. None of the surveyed employees classified themselves as symptomatic at entrance triage. However, three test-negative and two test-positive workers did report mild symptoms in our questionnaire (Table 1). At T2, one sewage sample tested positive (Ct value of 39 corresponding to approximately 5.5 copies/mL sewage).

**TABLE 1:** Characteristics of 76 Meat Processing Workers Participating in Naso-Oropharyngeal SARS-CoV-2 RNA Screening Performed on June 15, 2020

	*N*	SARS-CoV-2 Negative *N* = 49 (64.5%)	SARS-CoV-2 Positive *N* = 27 (35.5%)
Cooled production room			
Cutting	38	30 (79%)	8 (21%)
Deboning	38	19 (50%)	19 (50%)
Nationality			
Hungarian, Lithuanian, Portuguese, or Slovak	8	8 (100%)	0 (0%)
Polish	15	9 (60%)	6 (40%)
Romanian	53	32 (60%)	21 (40%)
Current residential situation			
Alone	13	8 (62%)	5 (38%)
Shared with up to four housemates	41	26 (63%)	15 (37%)
Shared with five or more housemates	19	13 (68%)	6 (32%)
Mode of transportation to work			
Alone (car/bike)	29	20 (69%)	9 (31%)
By public transport	3	2 (67%)	1 (33%)
By car/minivan with other people	42	25 (60%)	17 (40%)
Current province of residence			
Noord-Brabant (NL)	68	44 (65%)	24 (35%)
Nordrhein-Westfalen (DE)	8	5 (63%)	3 (37%)
COVID-19–related symptoms^a^ (*n* = 74)			
Without symptoms (self-reported)	69	44 (64%)	25 (36%)
With symptoms (self-reported)	5	3 (60%)	2 (40%)
Chronic disease status^b^ (*n* = 74)			
Without chronic condition (self-reported)	71	45 (63%)	26 (37%)
With chronic condition (self-reported)	3	2 (66%)	1 (33%)

Per characteristic, the number of participants is noted for which this data was available.

^a^Self-reported potential COVID-19–related symptoms included runny nose and loss of smell and/or taste (one worker tested positive and one worker tested negative), fever or feeling warm and loss of smell and/or taste (one worker tested negative), headache (one worker tested negative), and having a cough maybe/don't know (one worker tested positive).

^b^Chronic disease status defined as positive answer to the question “Do you have a chronic disease?” The person with chronic disease testing SARS-CoV-2 RNA positive reported hypertension controlled by β-blockers.

### Air and Surfaces

In total, 271 samples were collected (Table 2). At T2, SARS-CoV-2 RNA was detected in 9.8% of the surface swabs (6/61, Ct values of 38 to 39 corresponding to approximately 8 × 10^1^ to 1.6 × 10^2^ copies per swabbed surface). Of the 22 surface swabs collected at the cutting room at T2, three (14%) swabs tested positive, taken from a machine handle (with ridges), grip side of a stepladder, and the handle of a pressure pump used for disinfection. Of the 18 surface swabs collected at non-production areas at T2, three (17%) tested positive: swabs taken from a touch screen on the coffee machine, main touch screen for lockers in a changing room, and handle of a dispenser used for hand disinfecting. All six positive surfaces can be classified as high-touch. All 21 surface swabs collected in the deboning room at T2 were negative. All 142 surface swabs collected at T1 and T3 in production rooms as well as non-production areas were negative.

**TABLE 2:** SARS-CoV-2 RT-qPCR Test Results of a Total of 275 Samples Taken of Air, Surfaces, Workers' Hands, and Sewage in a Meat Processing Plant

Sampling Time Point	Sample Type	% Positive Samples (*n* Positives/*N*)
T1	Inhalable dust: stationary	0% (0/10)
T1	EDC	0% (0/16)
T1	Surface swab	0% (0/68)
T1	Sewage water	0% (0/2)
T2	Inhalable dust: stationary	0% (0/4)
T2	Inhalable dust: personal	8.3% (1/12)
T2	EDC	0% (0/6)
T2	Surface swab	9.8% (6/61)
T2	Sewage water	50% (1/2)
T2	Ventilation system filter	0% (0/8)
T2	Swab of hand worker	0% (0/12)
T3	Surface swab	0% (0/74)

T1 = June 8, 2020; T2 = June 15, 2020; T3 = June 19, 2020.

EDC, electrostatic dustfall collector.

SARS-CoV-2 RNA was detected in one of the 12 personal air samples (Ct value of 38 corresponding to approximately 5 × 10^2^ copies/m^3^). The worker with the SARS-CoV-2–positive air sample tested oro-nasopharyngeal positive at the start of the shift (Ct value: E gene, 33.2; RdRp gene, 33.8) but tested negative post-shift. Of the other 11 workers participating in the personal air sampling, one worker had a positive pre-shift and post-shift test (Ct values: E gene, 34.9 and 32.8, respectively; RdRp gene, 33.7 and 33.6, respectively); five workers only had a positive pre-shift swab (range in Ct values: E gene, 33.5 to 35.6; RdRp gene, 31.7 to 33.6; two, >40). SARS-CoV-2 RNA was not detected in any of the stationary inhalable dust samples (T1, *n* = 10; T2, *n* = 4). All other sample types (settling dust, filters ventilation system, swabs of workers' hands) also tested negative.

### Observations

Most of the 100 scored workers wore a surgical mask covering solely the mouth (66%, 29/40 cutting workers; 75%, 30/40 deboning workers; 55%, 11/20 packaging workers); others wore the mask covering mouth and nose. One person (deboning area) did not wear a mask. All of the 12 personal air sampling participants wore a mask; 11 (92%) wore the mask covering solely the mouth. Of the 11 personal air sampling participants with a negative air sample, nine had a stationary job task and few persons passed by their fixed positions along the line (most kept a 1.5-m distance). Seven of them worked at a position with eight or more persons working in 10-m vicinity; the other two workers were surrounded by respectively two and four persons. The two workers with non-stationary tasks showed frequent passing by or being passed by within a 1.5-m distance (several times per minute). The only worker with a positive personal air sample had a stationary job task in the deboning room and was surrounded by 10 persons in 10-m vicinity with a distance of >1.5 m from the nearest worker. Observations of personal air sampling participants were similar to 10 randomly selected workers per production room with respect to surrounding workers and 1.5-m distancing.

## DISCUSSION

Our findings provide insight into environmental contamination of SARS-CoV-2 in a large meat processing plant where comprehensive COVID-19 preventive and mitigation measures were already in place. Screening of workers' SARS-CoV-2 status by oro-nasopharyngeal swabbing showed a considerable percentage of workers to be SARS-CoV-2 RNA positive, with a relatively low viral load and generally without symptoms. Results of environmental sampling showed a low number of SARS-CoV-2 RNA-positive samples: one personal air sample and six frequently touched surfaces. This limited contamination of air and surfaces in both the cooled production rooms and non-production areas suggests SARS-CoV-2 environmental transmission to be under control in this plant during the period of our study.

### SARS-CoV-2 Status of Workers

Our investigation showed that one third of the workers tested positive for SARS-CoV-2 RNA in at least one of the two oro-nasopharyngeal swabs collected pre- and post-shift. Viral loads detected in the swabs were low, and workers were predominantly asymptomatic. There are several hypotheses to explain these findings: (1) worker(s) may have experienced a (mild) infection in the past without noticing/recalling symptoms (post-infection scenario), (2) worker(s) could be in pre-symptomatic state at the time of sampling (pre-symptomatic scenario), and (3) worker(s) could experience an asymptomatic infection (asymptomatic scenario). Published meta-analyses on SARS-CoV-2 strains circulating early in the pandemic reported percentages of SARS-CoV-2–infected persons remaining asymptomatic throughout infection of around 15% to 20%.^23–25^ Although percentages can be higher as observed in specific settings like single-family clusters (95% confidence interval, 26% to 44%)^24^ and certain occupational populations^26^ including German meat plant workers.^27^ SARS-CoV-2 RNA can remain detectable in swabs from the upper respiratory tract a couple of weeks after onset of infection.^28^ As workers who tested positive were followed up and no clear symptoms suggestive of COVID-19 had developed, the pre-symptomatic scenario seems unlikely. This leaves both occurrence of post-infections and asymptomatic infections as realistic. If we consider low RNA loads in participating workers a proxy of viral excretion,^29–31^ high shedding rates of SARS-CoV-2 for the majority are not to be expected; however, there might be individual differences and shedders among workers who were not tested. Most workers tested positive only pre-shift, which may be explained by physiological accumulation of respiratory tract secretions at the start of the day,^32^ swabbing differences between testers,^33^ and/or influence of stochastic processes especially at low viral loads (higher chance of false negatives). SARS-CoV-2 RNA level in the positive sewage sample was comparable with levels detected at urban sewage sites in the Netherlands in the early stage of the epidemic (March 2020).^34^ Because of site-to-site dissimilarities and methodological differences,^34,35^ the exact prevalence of infected workers cannot be estimated. These results of SARS-CoV-2 screening of workers emphasize the importance of mitigation measures in the workplace in light of asymptomatic infections on the one hand, but also as entrance triage appeared not fully effective in preventing persons with potential COVID-19–related symptoms to go to work.

### Environmental Contamination in Context

In the context of comprehensive prevention and mitigation measures in place at the time of the study, findings indicated absence of considerable SARS-CoV-2 levels in air throughout the cooled production areas. None of the stationary air samples were positive, despite the selection of likely hotspots. Central ventilation system filters were also all negative, whereas it has been suggested that SARS-CoV-2 RNA may accumulate in filters.^36^ Proper ventilation and cleaning of the ventilation system might have been of influence as also suggested in literature.^10,11,37^

One of 12 personal air samples was positive, with a 100-fold lower level than personal exposure levels measured in SARS-CoV-2–infected mink farms.^18^ As the Ct value of this air sample was too high for whole genome sequencing, and this worker's oro-nasopharyngeal swab tested positive, it could not be determined whether SARS-CoV-2 RNA detected in this personal air sample originated from this individual and/or from other workers. Low or non-detectable exposure as found in personal air samples can be explained by COVID-19 measures in place^38^ (eg, physical distancing, masks) and limited viral shedding by workers in line with low viral loads in oro-nasopharyngeal screening and negative personal air samples for six positive-tested workers. Inhalation exposure during a workday to such low/non-detectable levels of SARS-CoV-2 RNA (and even lower levels of viable virus) is not expected to pose a high risk of infection.^39^ Deposition of inhaled SARS-CoV-2 contaminated particles anywhere along the respiratory tract, from nasal epithelial cells to deep in the airways, has the potential to initiate infection,^40^ so air sampling covered the relevant particle size fraction.

The many surfaces sampled showed limited SARS-CoV-2 surface contamination, with low viral RNA loads in a few positive samples. As the hygiene standards in the food processing industry are high,^41,42^ regulations are already in place to ensure frequent and proper hand washing and disinfecting. This was substantiated by swabs from workers' hands/gloves being all negative for SARS-CoV-2 RNA. Given the sampling design—focusing on major high-touch surfaces and sampling later during the day so both shifts have passed—it is unlikely that the highest levels of surface contamination have been missed. Pork carcasses or meat products as a possible source can be excluded, as animal studies showed that pigs are unlikely to get infected with SARS-CoV-2.^43–45^ Considering limited SARS-CoV-2 RNA surface contamination observed (thus even lower considering viable virus), and focus on hand hygiene is in place, we consider this not a main route of transmission in this meat processing plant during the study period. This is in line with other real-life settings investigated for viral contamination of surfaces.^28^ To further minimize the risk, even more intense cleaning could be recommended for exceptionally high-touch surfaces in the non-production rooms (touch screens and handle) and non-smooth surfaces in the production rooms (handles/grip side).

### Comparisons to Other Research on Meat Processing Plants

In several countries worldwide,^9–12,27,46^ meat processing plants have been researched typically by outbreak investigations involving questionnaires and sampling of workers. The reported COVID-19 policies of the meat processing plants^9–12,27,46^ were a combination of measures directed at mitigating direct transmission and transmission via air and fomites. Quantifying the risk (absolute and relative) of each transmission route remains elusive especially as it will differ between facilities (eg, due to differences in layout, ventilation system, and airflow) and also will be varying over time per facility (eg, due to differences in measures implemented, human behavior, and viral strains involved). Overall, research indicated that strict COVID-19 prevention and mitigation measures were necessary to control outbreaks in investigated meat processing plants.^10,11,14,46^ The modeling study by Sobolik et al^14^ demonstrated that effective control could be obtained by bundled measures such as physical distancing, mask usage, increased ventilation, hand washing, and surface disinfection, resulting in a low risk of transmission for all routes between an infected worker and a susceptible worker. This is in line with our study findings showing limited environmental contamination in the presence of infected workers.

### Limitations

Sampling was performed during a 2-week period when a strict COVID-19 policy was in place. Because of this timing, no insight was gained into environmental contamination in an earlier stage or pre- and post-intervention comparisons. The total number of workers in the acute phase of infection (and thus shedding) remains unknown, as results of sewage screening are only indicative and oro-nasopharyngeal screening with RT-qPCR testing was performed in a subset of workers. Because of these limitations, general inferences on attributable effects of specific measures on the potential role of environmental transmission cannot be made.

As the level of environmental contamination was unknown before sampling, we focused on SARS-CoV-2 RNA detection to increase sensitivity and did not target specifically for viable virus. Because of low levels of viral RNA, viability testing could not be performed, and no inferences on potential levels of infectious virus were made. Finally, elucidating all potential transmission routes also outside the workplace was beyond the scope of this study.

### Outlook

Environmental sampling requires intense efforts and rapid action but is essential in providing empirical evidence. Measurements of (airborne) SARS-CoV-2 in occupational environments have been predominantly performed in hospitals and few other workplaces, but our study is the first to measure SARS-CoV-2 in meat processing plants.^16^ Setting up a comprehensive sampling campaign very rapidly in a non-stop operational facility is a daunting task and requires good cooperation between dedicated stakeholders. Looking beyond ad-hoc sampling campaigns, it would be interesting to explore options for routine monitoring (eg, implemented in ventilation systems) for (indicators of) SARS-CoV-2 and other pathogens. This pandemic clearly showed occupational populations like these to be facing multiple risk factors for communicable disease in general.^1,2^ COVID-19 policies to protect workers' health should be evaluated properly related to effectiveness and user-friendliness for the specific occupational context. For instance, in cooled production rooms, standard surgical masks can cause discomfort/annoyance as glasses fog easily and masks typically become moist quickly deteriorating effectiveness,^47,48^ emphasizing the need for research on measures in real-life settings.

## CONCLUSION

To conclude, given the overall low number of environmental samples positive for SARS-CoV-2 RNA, widespread transmission of SARS-CoV-2 via air and surfaces within this meat processing plant was not considered likely at the time of investigation when a strict COVID-19 policy was in place. This empirically substantiates that SARS-CoV-2 transmission can be controlled in meat processing plants with a comprehensive set of preventive and mitigation measures. The COVID-19 pandemic highlighted the vulnerability of this occupational population for infectious diseases and warrants for proper protection and monitoring.

## Supplementary Material

SUPPLEMENTARY MATERIAL
