# Supplementary material for: A Comprehensive Sampling Study on SARS-CoV-2 Contamination of Air and Surfaces in a Large Meat Processing Plant Experiencing COVID-19 Clusters in June 2020
Source: J Occup Environ Med. 2023 Jan 11;65(4):e227–33. doi: 10.1097/JOM.0000000000002785 (PMC10090283; doi:10.1097/JOM.0000000000002785)
Supplement: SUPPLEMENTARY MATERIAL [file joem-65-e227-s003.docx]

# Supplemental Digital Content accompanying manuscript

**A comprehensive sampling study on SARS-CoV-2 contamination of air and surfaces in a large meat processing plant experiencing COVID-19 clusters in June 2020**

## Supplemental Methods

**Additional information on the investigated meat processing plant**

Cooled production-associated areas are solely accessible for workers operating in the cooled production rooms. These include a canteen area with restaurant, various changing rooms with lockers and toilet facilities, passageways with staircases and one large hygiene lock. Areas are cleaned daily and toilet facilities cleaned twice a day with designated cleaning/disinfecting agents including chlorine-based agents.

Face shields were solely worn by workers with communication duties (e.g. foremen/intendents) in line with the slaughterhouse’s policy.

**Additional information on sampling**

*Oro-nasopharyngeal sampling*

Oro-nasopharyngeal sampling was performed according to the June 2020 prevailing national monitoring protocol (<https://lci.rivm.nl/richtlijnen/covid-19>); the same swab was used to first swab the oropharynx followed by the nasopharynx. Thereafter, the swab was directly placed in 3 ml GLY virus transport medium.

*Questionnaires*

Self-reported data obtained from employees was collected using questionnaires focusing on the following topics: current and prior symptoms, overall health and chronic conditions, living situation, contact with workers in meat-processing facilities, contact with COVID-19 cases, recent travel history, work situation and workplace, contact with co-workers, and commuting. The questionnaires were available in five languages (Dutch, English, Polish, Hungarian and Romanian). Questionnaires were distributed upon the first sampling and collected upon the second sampling.

*Personal and stationary air sampling*

Teflon filters (Pall Corporation, Ann Arbor, USA) were used in GSP (Gesamtstaubprobenahme, total dust sampling; JS Holdings, Stevenage, UK) sampling heads connected to a Gilian GilAir 5 pump (Sensidyne, St. Petersburg, USA) calibrated at a flow of 3.5 l/min.

Picture showing stationary air sampling (orange circle around sampling head):

**
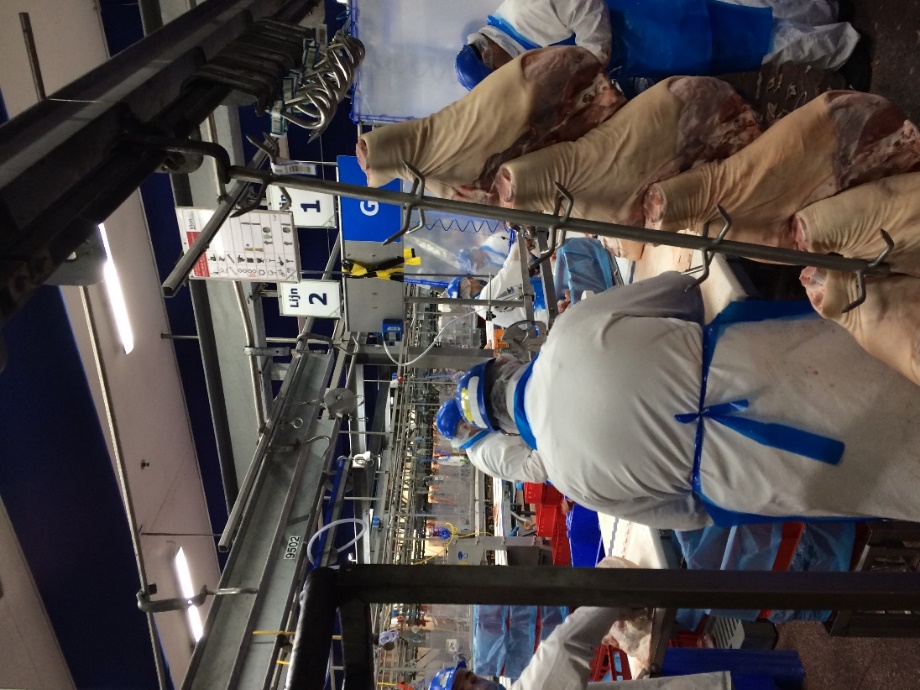
**

Pictures showing personal air sampling:


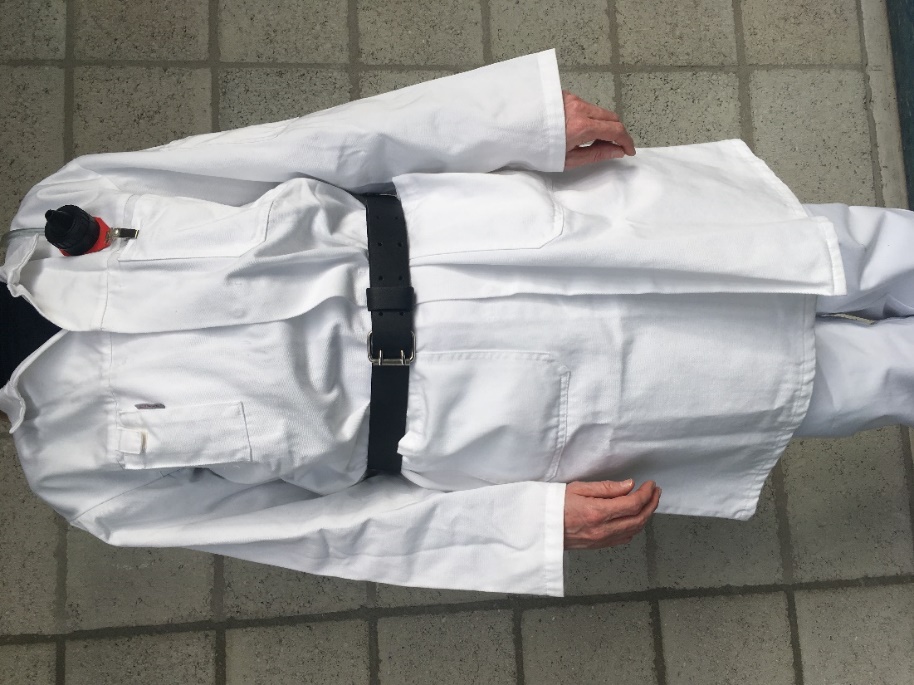

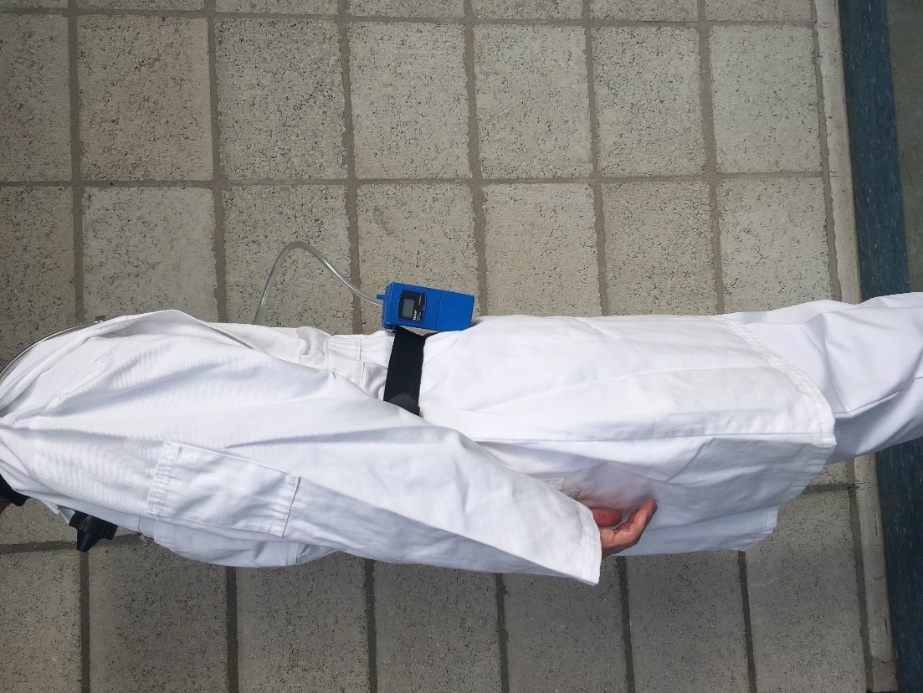


Note. Due to privacy reasons, a picture of a researcher was taken and not of a slaughterhouse worker

*Electrostatic Dust fall Collectors (EDCs)*

EDCs are sterilized electrostatic cloths (polyester electrostatic cloth; Albert Heijn, Zaandam, the Netherlands) placed in a disposable holder. At T1, 5 EDCs were placed per production room; these could only be exposed during one day due to the cleaning regime involving bottom-to-top wetting. EDCs placed in the canteen area were exposed during 7 days. At T1, 6 EDCs were placed in the canteen area, which were collected at T2 and replaced with new EDCs which were collected at T3.

Picture showing EDC positioned on top of machine in cutting room:

**
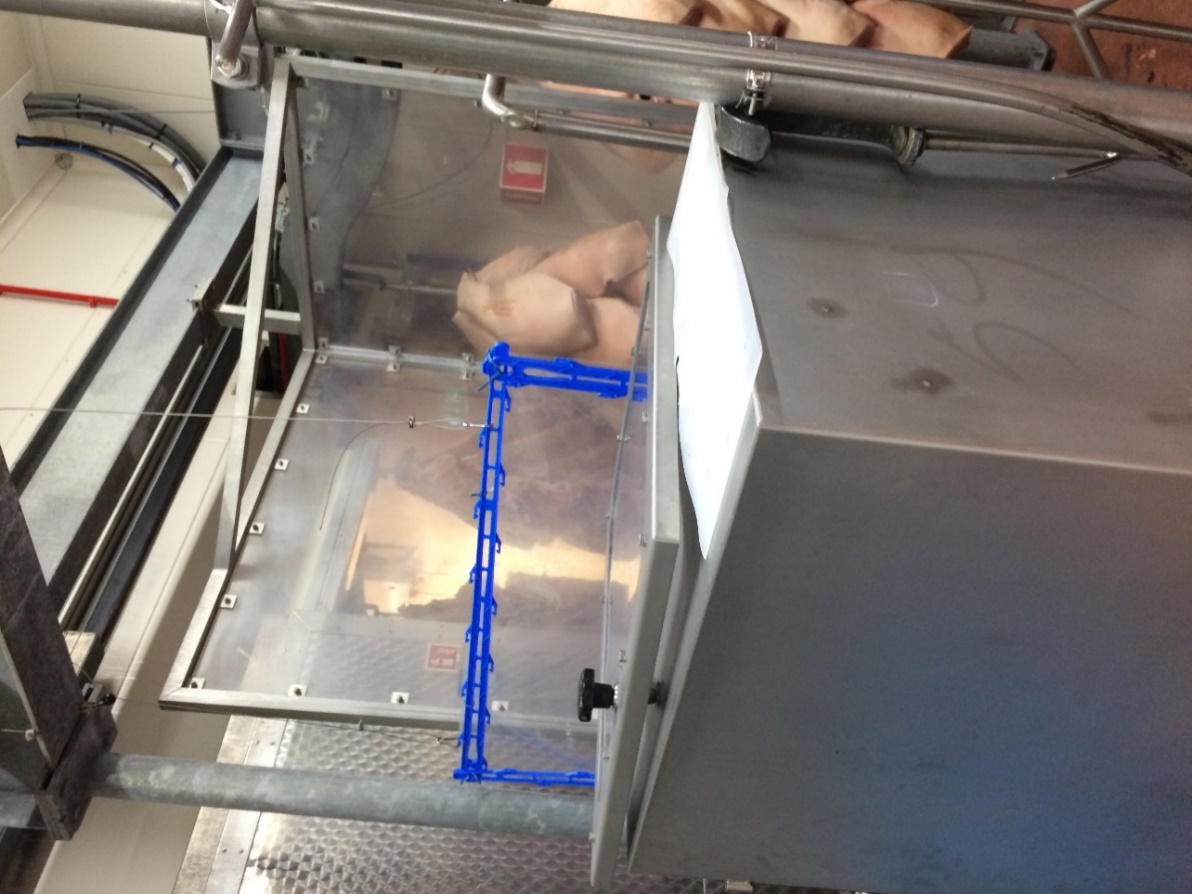
**

*Surface swabs*

Swabbed items included knobs, grips, push buttons, touchscreens and all sorts of handles (e.g. machinery in production rooms, dispensers in toilets); but also table tops, chairs, stair railings and other surfaces frequently touched. Disposable plastic grids of 10 cm^2^ were used for standardization of the sampled surface. If a surface was smaller than 10 cm^2^ this was noted. Dry swabs with a rayon tip and plastic shaft (CLASSIQSwabs 167KS01; COPAN, Brescia, Italy) were used, which were placed in 2ml virus transport medium (VTM) directly after swabbing.

*Swabs of workers’ hands/gloves*

Dry swabs with a rayon tip and plastic shaft (CLASSIQSwabs 167KS01; COPAN, Brescia, Italy) of hands, or gloves if worn, of the 12 workers participating in the personal air sampling were collected during their mid-shift break. The inner part of their index finger, middle finger and ring finger was swabbed until the half of their palm.

*Specifications of fieldworkers and field blanks*

Fieldworkers that visited the meat processing plant for sampling were routinely monitored for SARS-CoV-2 infection (remained negative throughout the study). Field blanks of all sample types were collected as a control, these blanks underwent all procedures (e.g. preparations, transportation, processing) as the actual samples except for sampling. All field blanks tested negative (no curve, more than 42 cycles run).

**Details on laboratory analyses**

*Electrostatic Dust fall Collectors (EDCs)*

One tablet of protease inhibitor was dissolved in 20mL D-PBS, without Calcium and Magnesium. Half of the EDC/mouth mask was put into a 50mL tube, containing 10 mL D-PBS+protease inhibitor and incubated for 1 hour at room temperature on a tube roller. 60uL sample was removed, and 90 ul MagNA Pure 96 External Lysis Buffer (Roche) was added. PDV (10 uL) was used as internal control, as described previously^40^. RNA was eluted in 30 uL distilled water, 8 uL was used for the SARS-CoV-2 PCR, as described previously^17^.

*Surface swabs and swabs of workers’ hand/gloves*

Swabs in 1 ml VTM were vortexed, and 60 uL VT was further processed as described above.

*Air filters (Teflon and ventilation system)*

Teflon filters were collected from the GSP sampling heads and transferred to a 15ml tube. Per ventilation system filter, 2 punches were taken (25mm diameter, approximately 6mm thick). Each punch was transferred to a 15ml tube. To each tube 1ml VTM and 1 ml lysisbuffer (MagNA Pure 96 External, Roche) was added and subsequently vortexed for 5 minutes. PDV (10uL) was added to 150 ul of sample. RNA extraction and SARS-CoV-2 RT-PCR was performed as described above.

*Sewage*

Each tube containing 50 ml of sewage was spinned down (3000g during 15 min) and 15 ml of the supernatant was transferred to an Amicon tube. The sample was subsequently spinned down during 30 min at 4000g. The filter was rinsed with PBS and transferred into a new tube. RNA extraction and SAR-CoV-2 RT-PCR was performed as described above.

*Schematic overview of PCR analysed proportion per sample type*

| Sample type | Original sample | Volume of medium added (ml) | Volume aliquot of sample for PCR (µl) | Volume added to aliquot (µl) | Volume eluens (µl) | Volume in PCR (µl) | Proportion volume PCR/volume eluens | Proportion volume aliquot/sample volume medium | Proportion subsample of original sample |
| --- | --- | --- | --- | --- | --- | --- | --- | --- | --- |
| Teflon filter T2 (filter used in active air sampling) | 1 filter ~ cubic meter of air sampled dependent on flow rate and sampling duration | 2 ml (1 ml VTM + 1 ml lysisbuffer) | 150 🡪 75 µl sample | 10 µl PDV | 30 | 8 | 0.267 | 0.075 | 0.00415 |
| Swab T2 | 1 swab ~ swabbed surface of max 10 cm^2^ | 1 ml VTM | 60 | 100 µl (90 µl lysis buffer + 10 µl PDV) | 30 | 8 | 0.267 | 0.06 | 0.01602 |
| Sewage sample T2 | 15 ml out of 50 ml tube concentrated to 200 µl ~ 24 h flow-dependent composite sample | None | 60 | 100 µl (90 µl lysis buffer + 10 µl PDV) | 30 | 8 | 0.267 | 0.3 | 0.0801 |
